# Supplementary material for: Associations between Body Mass Index, Waist Circumference, and Myocardial Infarction in Older Adults Aged over 75 Years: A Population-Based Cohort Study
Source: Medicina (Kaunas). 2022 Nov 30;58(12):1768. doi: 10.3390/medicina58121768 (PMC9783624; doi:10.3390/medicina58121768)
Supplement: Supplementary file 1 [file medicina-58-01768-s001.zip › medicina-2014437-supplementary.pdf]

Supplementary Material

Supplementary Table S1. Hazard ratio and 95% confidence intervals for MI according to BMI and WC 5 levels and hypertension, DM, hyperlipidemia using model 5 (Cox's proportional hazard model)

BMI	N	MI	Hazard Ratio (95% CI)
Group 10	18,543	576	1.189 (1.085,1.304)
Group 11	38,150	1,839	2.063 (1.938,2.197)
Group 20	72,236	2,196	1 (Ref.)
Group 21	241,604	11,687	1.703 (1.627,1.783)
Group 30	26,007	780	0.932 (0.858,1.011)
Group 31	148,813	7,453	1.655 (1.577,1.737)
Group 40	19,089	557	0.902 (0.821,0.991)
Group 41	174,588	8,484	1.581 (1.505,1.661)
Group 50	1,072	30	0.909 (0.634,1.305)
Group 51	18,369	820	1.471 (1.352,1.600)
**WC**	**N**	**MI**	**Hazard Ratio (95% CI)**
Group 10	10,650	327	0.999 (0.886,1.126)
Group 11	18,736	842	1.665 (1.530,1.812)
Group 20	46,381	1,381	0.947 (0.882,1.018)
Group 21	119,496	5,635	1.631 (1.545,1.723)
Group 30	56,590	1,727	1 (Ref.)
Group 31	267,017	13,018	1.725 (1.640,1.814)
Group 40	20,541	616	1.045 (0.952,1.146)
Group 41	174,178	8,713	1.874 (1.776,1.978)
Group 50	2,785	88	1.210 (0.976,1.501)
Group 51	42,097	2,075	2.030 (1.893,2.176)

Model 5: adjusted for age, sex, smoking, heavy drinking, physical activity, low income, COPD, cancer, BMI, and WC

Group 10: level 1 & TDL 0
Group 20: level 2 & TDL 0
Group 30: level 3 & TDL 0
Group 40: level 4 & TDL 0
Group 50: level 5 & TDL 0
Group 11: level 1 & TDL 1
Group 21: level 2 & TDL 1
Group 31: level 3 & TDL 1
Group 41: level 4 & TDL 1
Group 51: level 5 & TDL 1

TDL 0: subject with no hypertension (T), diabetes (D), or hyperlipidemia (L)
TDL 1: subject with one or more of hypertension (T), diabetes (D), or hyperlipidemia (L)
BMI, body mass index; MI, myocardial infarction; WC, waist circumference; DM, diabetes mellitus

Supplementary Table S2. Hazard ratio and 95% confidence intervals for MI incidence according to segmented sections of BMI and WC using model 5 (Cox's proportional hazard model)

**BMI_seg**	**N**	**MI**	**Hazard Ratio (95% CI)**
BMI< 16	19,385	803	1.376 (1.273,1.486)
16≤ BMI< 17	21,383	955	1.282 (1.193,1.378)
17≤ BMI < 18	35,382	1,471	1.110 (1.044,1.179)
18≤ BMI < 19	51,920	2,349	1.134 (1.077,1.195)
19≤ BMI < 20	69,457	2,956	1.006 (0.958,1.055)
20≤ BMI < 21	84,585	3,790	1 (Ref.)
21≤ BMI < 22	88,421	3,974	0.948 (0.907,0.991)
22≤ BMI < 23	91,995	4,386	0.974 (0.932,1.018)
23≤ BMI < 24	82,825	3,847	0.918 (0.877,0.961)
24≤ BMI < 25	68,759	3,171	0.890 (0.848,0.934)
25≤ BMI < 26	52,044	2,429	0.884 (0.839,0.932)
26≤ BMI < 27	35,715	1,673	0.870 (0.820,0.924)
27≤ BMI < 28	23,186	1,115	0.889 (0.830,0.953)
28≤ BMI < 29	13,973	653	0.854 (0.784,0.930)
29≤ BMI < 30	8,179	351	0.778 (0.696,0.870)
30≤ BMI < 31	4,617	212	0.834 (0.725,0.960)
31≤ BMI < 32	3,125	140	0.813 (0.685,0.964)
32≤ BMI	3,520	147	0.758 (0.641,0.896)

**WC_seg**	**N**	**MI**	**hazard ratio (95% CI)**
M<70/F<65	29,386	1,169	0.976 (0.913,1.043)
70 ≤ M < 75/ 65 ≤ F <70	57,798	2,417	0.955 (0.908,1.004)
75 ≤ M < 80/ 70 ≤ F <75	108,079	4,599	0.945 (0.909,0.983)
80 ≤ M < 85/ 75 ≤ F <80	153,946	6,911	0.973 (0.941,1.005)
85≤ M < 90/ 80 ≤ F <85	169,661	7,834	1 (Ref.)
90 ≤ M < 95/ 85 ≤ F <90	124,020	5,821	1.018 (0.984,1.054)
95 ≤ M < 100/ 90 ≤ F <95	70,699	3,508	1.111 (1.065,1.158)
100≤ M < 105/ 95 ≤ F <100	30,082	1,430	1.089 (1.026,1.156)
M≥105/F≥100	14,800	733	1.216 (1.121,1.319)

BMI, body mass index; MI, myocardial infarction; WC, waist circumference

Model 5: adjusted for age, sex, smoking, heavy drinking, physical activity, low income, COPD, cancer, BMI, and WC
